# Strategies for adapting under pressure: an interview study in intensive care units

**DOI:** 10.1136/bmjqs-2024-017385

**Published:** 2024-08-23

**Authors:** Bethan Page, Dulcie Irving, Jane Carthey, John Welch, Helen Higham, Charles Vincent

**Affiliations:** 1 Department of Experimental Psychology, University of Oxford, Oxford, Oxfordshire, UK; 2 Cicely Saunders Institute, King's College London, London, Greater London, UK; 3 Human Factors and Patient Safety, Jane Carthey Consulting, Chiswick, UK; 4 National Institute for Health and Care Research Central London Patient Safety Research Collaborative, University College London Hospitals NHS Foundation Trust, London, UK; 5 Nuffield Department of Clinical Neurosciences, University of Oxford, Oxford, UK; 6 Nuffield Department of Anaesthetics, Oxford University Hospitals NHS Foundation Trust, Oxford, UK

**Keywords:** Risk management, Critical care, Qualitative research, Leadership, Crisis management

## Abstract

**Background:**

Healthcare systems are operating under substantial pressures. Clinicians and managers are constantly having to make adaptations, which are typically improvised, highly variable and not coordinated across teams. This study aimed to identify and describe the types of everyday pressures in intensive care and the adaptive strategies staff use to respond, with the longer-term aim of developing practical and coordinated strategies for managing under pressure.

**Methods:**

We conducted qualitative semi-structured interviews with 20 senior multidisciplinary healthcare professionals from intensive care units (ICUs) in 4 major hospitals in the UK. The interviews explored the everyday pressures faced by intensive care staff and the strategies they use to adapt. A thematic template analysis approach was used to analyse the data based on our previously empirically developed taxonomy of pressures and strategies.

**Results:**

The principal source of pressure described was a shortage of staff with the necessary skills and experience to care for the increased numbers and complexity of patients which, in turn, increased staff workload and reduced patient flow. Strategies were categorised into anticipatory (in advance of anticipated pressures) and on the day. The dynamic and unpredictable demands on ICUs meant that strategies were mostly deployed on the day, most commonly by flexing staff, prioritisation of patients and tasks and increasing modes of communication and support.

**Conclusions:**

ICU staff use a wide variety of adaptive strategies at times of pressure to minimise risk and maintain a reasonable standard of care for patients. These findings provide the foundation for a portfolio of strategies, which can be flexibly employed when under pressure. There is considerable potential for training clinical leaders and teams in the effective use of adaptive strategies.

**WHAT IS ALREADY KNOWN ON THIS TOPIC**

**WHAT THIS STUDY ADDS**

- This study empirically presents a menu of strategies used in intensive care for adapting under pressure gathered through interviews with different professionals involved in patient care and the running of the service.

**HOW THIS STUDY MIGHT AFFECT RESEARCH, PRACTICE OR POLICY**

- These findings could be used as the basis of training programmes for intensive care units to develop a set of coordinated strategies for adapting under pressure.

## Introduction

 Health services have been under increasing pressure for many years as the result of an ageing population, increasing complexity of illnesses and comorbidities, as well as a shortage of resources of all kinds (particularly skilled and experienced staff). The intensive care unit (ICU) is a particularly pressurised environment that requires frequent adaptation to maintain patient safety when placed under pressure.^1 2^ The aftermath of the recent COVID-19 pandemic saw many nursing staff exiting the National Health Service (NHS), leaving ICUs chronically short of skilled, experienced staff.^3 4^ Healthcare system pressures have tangible effects on the way work is done, staff experience and patient care.^5 6^

Clinical teams adapt to these everyday pressures to minimise risk to patients, and in most cases, achieve good outcomes despite adverse circumstances.^6^ While individual adaptations of care may be reasonable and necessary, the overall effect of multiple adaptations on the quality of care is variable.^7^ We have previously argued that clinical leaders need to employ a portfolio of strategies when pressures are high and develop a coordinated approach for teams to deliver safe care and service efficiency within available resources.^5^

Our previous review of empirical resilient healthcare studies explored the nature of adaptations to care at times of pressure and created a taxonomy of pressures and adaptations.^8^ This review built on earlier work on resilient healthcare and adaptive capacity.^8^,^13^ Our review found that the primary source of pressure is a mismatch between demand and capacity. Working conditions then become more difficult which in turn increases risk to patients and creates more pressure on staff. We found that adaptive strategies could be divided into actions taken in anticipation of pressures and actions taken on the day. The primary types of adaptive strategy, both in advance and on the day, concerned methods for increasing or flexing resources (e.g adapting staff skill mix), approaches to controlling demand and adaptations to the standard and manner in which care is delivered.

The studies included in the previous review contained useful descriptions of adaptations but are focused more on understanding and advancing resilience theory. This current paper seeks to describe strategies used by front-line clinicians so that they may be developed and used in a practical sense to help reduce risks to patient safety and burden on staff, rather than to further advance resilience theory. Only two studies included in our previous review examined intensive care.^14 15^ In the current study, we explore in depth how different teams in ICUs are adapting to everyday pressures and describe the adaptive strategies they use to respond, using the previously published taxonomy as a framework for the analysis. The aim of the present study is therefore to explore the everyday pressures experienced in multiple adult ICUs and to identify ways in which intensive care teams adapt clinical practice to meet demand while managing risks to patients.

## Methods

### Study design

Semistructured interviews were conducted with senior healthcare professionals (defined as those with line-management responsibility for a team and/or the running of a service) working in adult ICUs in four hospitals across England to identify the types of everyday pressures experienced and the adaptive strategies used. Reporting of the methods follows the Consolidated Criteria for Reporting Qualitative Research guidelines checklist for interviews and focus groups.

### Sampling and setting

Four acute hospitals from across England were purposively sampled to capture different geographical locations, size and type of hospital and population demographics. Three were large teaching hospitals with multiple ICUs split across different sites and one was a District General Hospital with a smaller ICU. As a collective, they provided adult general, surgery, cancer and haematology intensive care services. All ICUs had intensive care outreach services attached or a Medical Consultant assigned to outreach for each shift. Data collection was completed between September 2022 and October 2023.

We purposively sampled senior staff from across disciplines working in ICU (see definition above), as they have more autonomy and requirement to make system-level adaptations than more junior staff. We identified a lead contact known to the researchers in each hospital for consultation, suggestions of interviewees and communication of findings. We approached 25 members of ICU teams, with 20 agreeing to be interviewed and no response from five. After 20 interviews, it was agreed that data saturation had been reached. The final sample consisted of medical consultants (n=5, including one head of department), matron or nursing leads (n=9, including senior outreach staff and an ICU manager), senior nurses (n=4) and senior physiotherapists (n=2).

### Data collection

A semistructured interview guide was developed, informed by the findings from our recent scoping review on resilient healthcare^8^ and adapted in response to two pilot interviews (online supplemental file 1). Participants were invited to take part via email and sent a full information sheet. Verbal consent was obtained at the beginning of each interview, which included permission to record the interview for the purpose of generating a transcript. Interviews were conducted by a combination of two of the research team: a human factors and patient safety consultant (JC) and researchers experienced in qualitative methods and healthcare research (BP and DI). Semistructured interviews were conducted over video call, audio recorded and transcribed verbatim. Field notes were taken during the interviews to follow-up on points of interest or for clarification. Each interview lasted approximately 1 hour. No repeat interviews were conducted.

### Analysis

The data were analysed using a thematic template approach.^16 17^ The qualitative data management tool NVivo was used to manage and code the interview data. The first stage of analysis was data familiarisation: BP and DI read the interview transcripts and shared initial reflections and preliminary coding strategies during a series of meetings with other members of the research team (CV and JC). In the second phase, we created and iteratively developed a framework for organising the data, drawing on the interview guide and our previously developed taxonomies of pressures and strategies.^8^ The resulting coding framework provided the major theme headings for the analysis (online supplemental file 2). The framework was piloted on a sample of four interviews and was found to capture the key pressures and strategies described. Data from each transcript were then indexed through systematically coding quotations and placing them in one (or more) of the framework categories. BP and DI led the data analysis, and CV and JC provided oversight. The interpretation of the data was sense-checked by clinicians HH and JW. The research team met regularly to discuss the analysis and coding framework.

### Reflexivity and quality assurance

The researchers who conducted the interviews and led the data analysis were health services researchers with expertise and graduate degrees in the field of patient safety. The two coders independently coded the interviews and compared their results through discussion, providing assurance for the consistency of coding and interpretation. Emerging findings were discussed in regular research team meetings. The results were developed with input from two highly experienced clinicians (a consultant ICU nurse and a consultant anaesthetist) who supported the interpretation of the data, clarified clinical issues and which led to a deeper and more nuanced understanding of the data. The findings were presented and discussed with a wider group of front-line intensive care clinicians as a sense-check.

## Results

### Pressures

The pressures were broadly categorised into (1) demand exceeding capacity, (2) difficult working conditions and (3) problems with system functioning.^8^

**Figure 1:**
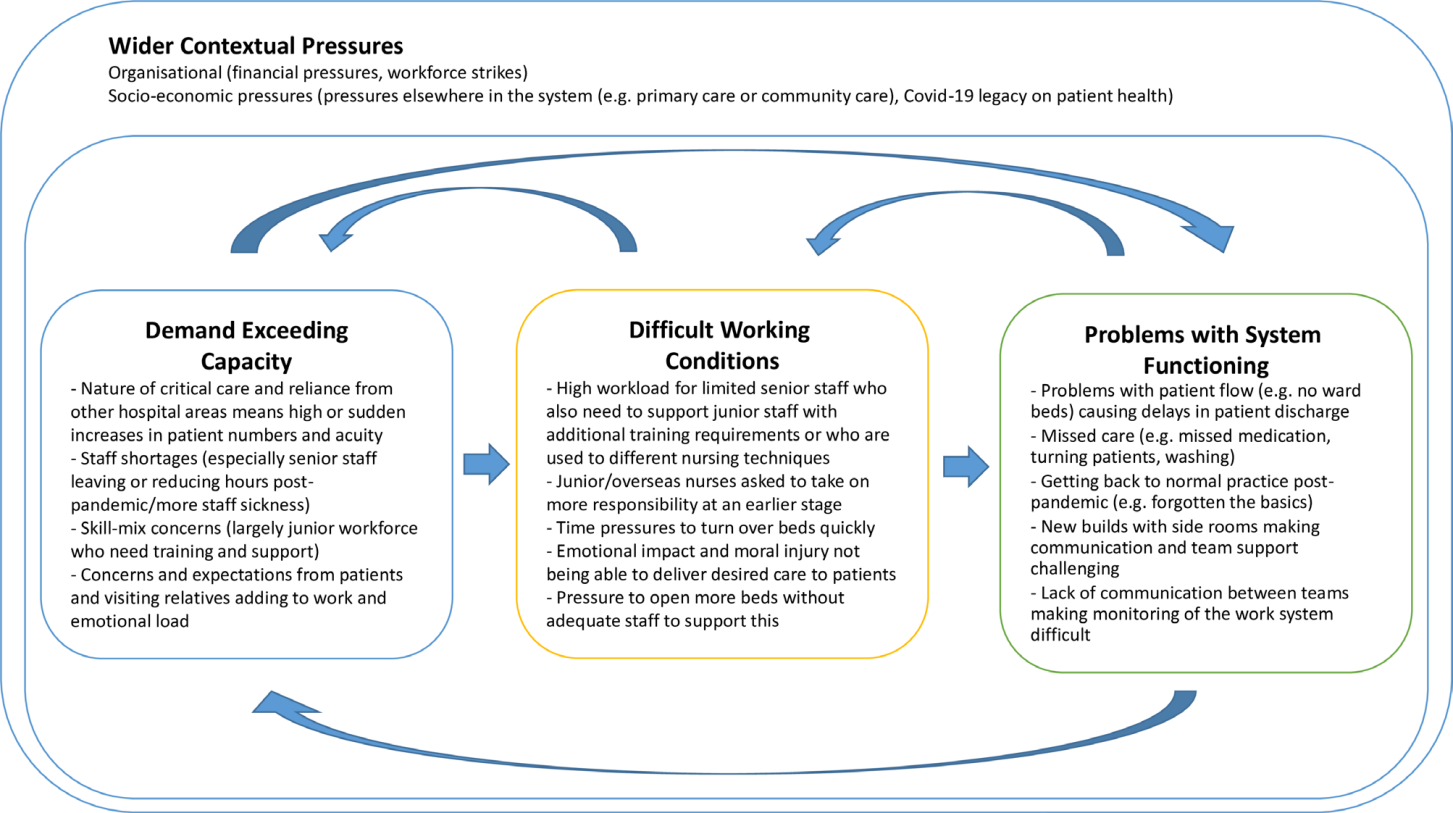
Interrelationships between pressures commonly experienced in intensive care.

Those interviewed reported that the gap between demand and capacity has steadily increased in recent years, particularly post pandemic, because of the shortage of skilled, experienced ICU nurses. This has created severe skill-mix problems, leaving a very junior workforce expected to take on more responsibilities at an earlier stage of their careers and increasing the workload for the senior nurses who remain. Bed shortages were commonly cited, but the primary difficulty is the shortage of nurses to staff those beds rather than physical capacity. There are in addition multiple interactions and feedback loops between the different pressures and problems. For example, shortages of skilled staff and workload pressures often results in delayed assessment, missed or delayed care (for instance, missed medication) leading to longer patient stays which in turn reduces capacity to cope with new patients coming into the system. Figure 1 demonstrates this dynamic process and summarises the principal pressures described by intensive care teams.

### Anticipatory strategies

In anticipation of increasing pressures, staff endeavoured to adjust resources to meet anticipated demand and make plans for managing the workload (see table 1 and box 1 for examples). Plans for controlling demand and adapting the delivery of care are typically made a few days in advance, whereas plans to increase resources tend to be longer term.

**Table 1:** Anticipatory strategies used in ICUs with illustrative examples

Theme	Anticipatory strategy	Example
**Increase resources**		
	Initiatives to increase staff in longer-term recruitment	Substantial numbers of international nursing graduates recruited to fill vacancies. A hospital employed a dedicated recruitment and retention nurse to support them. Non-clinical staff team recruited to manage stores (eg, restocking supplies), previously managed by nurses on shift.
	Strategies to create temporary capacity in the short-term	Relocate clinical skills practitioners from educational roles to work clinical shifts in order to bridge staff shortage gaps. Advertise for bank staff, often through social groups (eg, Facebook/WhatsApp). Some hospitals used agency staff on a regular basis and others only in emergencies.
	Improving skill mix of existing staff through additional training	Create specialist job roles to improve skill mix. For example, specialist nurses who are trained to treat deteriorating patients. Education and professional development of new international nursing graduates in clinical skills and non-technical skills, such as speaking up about concerns. Train healthcare assistants to notice deterioration in blood pressure to alert nurses, allowing nurses to do other work.
	Managers required to take on more clinical hours	Managers take on more clinical work themselves to address skill-mix concerns and cut back their managerial time.
**Control demand**		
	Flexing admission and discharge criteria depending on pressure in the unit	Use outreach teams to identify ward patients at risk of deterioration or by ‘weather-forecasting’ potential ICU admissions based on patient demand coming through the emergency department and acute medical unit. Discharge patients to ward care who would usually remain on the ICU for further days or transfer to another ICU/hospital in the network if needed. This was reciprocal based on fluctuating pressures in each hospital within the network. Pre-emptively admit patients to ICU to relieve pressure elsewhere (eg, emergency department).
	Discharging patients directly home from the ICU instead of to ward with follow-up support at home	Discharge patients straight home from intensive care rather than to a ward (if home support is available) and use outreach teams to follow-up. These patients would be carefully selected prior to discharge with the safety net of the outreach team there to be a point of contact for carers at home.
	Cancelling elective surgeries	Cancel elective surgery for patients likely to need an ICU bed following their surgery. This was generally a last resort.
**Plans for managing the workload**		
Efficiency strategies	Using technological aids to make communication more efficient	New communication device (eg, Vocera), “like a little Alexa”, for communicating with colleagues, helpful for feeling supported and saving time (eg, contacting lab directly for emergency blood). Use of an app for storing reports and sharing news, making it easier to access information and communicate updates.
Forward planning	Contingency planning and anticipating potential scenarios	Use morning safety huddles to have “what are we going to do if…” conversations and having a back-up plan for when an unusual event occurs (eg, paediatric admission). Double-up regular and agency staff on the rota to allow for on-the-day absences of either.
	Creating or adapting protocols to reinforce the basics	Create or simplify existing protocols to make them more accessible for junior staff and increase eligibility for tasks. Provide simulation training of protocols to remind staff of the essentials of care, and test out new/adapted protocols.
Monitoring and coordination strategies	Having planned meetings for monitoring the situation and communicating plans	Fixed checkpoint meetings (eg, safety huddles, sit reps) throughout the day to communicate emerging concerns and identify priorities with the team. These meetings always went ahead even when the units were under intense pressure.
	Having up-to-date knowledge of resources and demand in ICU and wider hospital	Keep track of available beds on the wards to discharge patients to and elective surgery patients who may need intensive care beds. Outreach teams were described as ‘the external sensors of ICU’ and helped to identify deteriorating patients on the ward who might need admitting to ICU.
	Centralised structures for co-ordination	One of the larger hospitals with multiple ICUs had introduced a new coordinator role (‘operational nurse in charge’) who communicated staff number and skill mix needs across each of the ICUs.
	Staff rotas organised to share difficult shifts evenly	Identify shifts with heavy/high acuity patient load and use of personal protective equipment so that such work could be evenly distributed between staff.
Staff support initiatives	Initiatives to provide support to staff	Dedicated wellness teams and allocated psychologists to provide emotional and practical support to staff. Professional nurse advocates with allocated time for the well-being of colleagues, including providing break relief, help with competencies or restorative supervision sessions. Additional support for international nursing graduates to help them transition including specialised induction programmes and buddy systems.

ICUintensive care unit

Box 1 **Anticipatory strategies in intensive care units**

**Increasing staff and opening services to increase capacity and reduce delays in care**

“So did a workforce review last year and pushed forward a business case to increase by an additional practitioner, which allowed me to open up weekend working. That’s made a significant difference to our day-to-day workflow. It now means that when we come in on a Monday, we’re not coming in to upwards of nine to ten brand-new patients that have been sitting greater than 48 hours waiting for an assessment. We’re coming in to patients that have been seen over the weekend and risks are reduced” (Outreach Clinical Lead)

**Creating or adapting protocols to help solve staffing skills and experience profile change post-pandemic**

“We've really gone into overdrive in developing more visual and easier to see protocols, SOPs [Standard Operating Procedure], that kind of thing. We were moving towards that anyway. It's definitely gone up a gear, the view that people need to have that stuff to remind them of essentials of care…now we have a much bigger set of nicely coloured photographs and illustrated guides. They're either laminated or put onto a phone system. We have an app that you can call up some of these protocols and guides. We've also linked the same products to the electronic patient records that people can call up there too. There's now a whole set of ways of accessing that stuff.” [Consultant Nurse]

**Planned meetings to monitor and coordinate contingency plans to prepare for sudden stresses on the system**

“So we have our safety huddle in the morning. We will have a what-are-we-going-to-do-if conversation. We may have a day when we feel quite flush with staff, but we always have that awareness that even if you have two more members of staff, if the emergency goes much more than the system can cope with, you’ve got to know what you’re going to do, which is your plan B. How are you going to split out your resources? And that doesn’t come down to just numbers of staff. It comes down to which staff you’ve got, and which skill set they’ve got.” [Outreach Clinical Lead]

#### Increase resources

Efforts to increase resources in intensive care mostly focused on increasing staffing and improving skill mix rather than on beds, equipment or supplies. There were large recruitment drives underway for international nursing graduates in two of the hospitals to help fill the vacancies and address increased demand. New job roles had been created in some units. For instance, a new non-clinical team had been introduced in one hospital to manage the stores, allowing nurses to focus on patient care. All hospitals had invested in education and professional development teams, with special attention given to supporting and enhancing the skills of new international nurses.

#### Control demand

Forecasting was used by most units to anticipate and control demand. For instance, teams would assess how stable the staffing rota looked in the next 24–48 hours before agreeing that they could safely admit a new patient. In some cases, units employed their outreach teams to identify patients on wards who were at risk of deterioration and admit them earlier if the ICU had capacity or to support their care on the ward if there was no ICU capacity. Identifying patients ready for discharge was a daily priority, for which one hospital had developed a traffic light dashboard system to reduce the cognitive load of monitoring the status of the unit and making these decisions.

The criteria for entering and being discharged from intensive care were constantly flexed depending on the degree of pressure. Clinicians assessed patients for discharge home or transfer to another hospital, considering available support, time of day and day of week, and family factors such as distance to visit. At times of very short staffing, or on occasions such as Christmas, some units closed parts of the ICU in order to concentrate the available resources in one area. Some units, setting a completely new precedent, had now begun to discharge patients directly home from the ICU to be followed up by the outreach team at home over phone call.

#### Plans for managing the workload

Clinical protocols were standardised and simplified to make them more accessible and usable by junior nurses, so that more senior nurses could be used to supervise nurses with less experience of intensive care. Multiple fixed meetings were held throughout the day to monitor resources, communicate concerns and identify priorities. Technological aids such as walkie-talkies were introduced to make communication between side rooms or requesting blood from the lab more efficient. Education and training were delivered where possible by incorporating them alongside clinical work (eg, ‘tea trolley teaching’).

### On-the-day adaptations

Forecasts and plans often changed on the day in intensive care, and so clinical teams had to be adaptable and amenable to change. Table 2 and box 2 illustrate strategies deployed to flex resources, prioritise demand and adapt ways of working on the day.

**Table 2:** On-the-day adaptations used in ICUs with illustrative examples

Theme	On-the-day adaptation	Example
**Flex resources**		
	Manage patients who need intensive care outside unit	Outreach team used to manage patients on the ward until bed becomes available in ICU. Provide care to patients in the emergency department to prevent further deterioration before delayed admission to ICU.
	Flexing staff to address numbers or skill mix	Adaptively flex staffing throughout the day based on acuity of patients, nursing ratios and available skill mix of staff. For example, replace junior team members with more experienced staff when patient’s acuity exceeds experience and skill set.
	Managerial staff take on clinical roles and responsibilities	Education team help on busy days or provide intermittent break relief. Senior nurses postpone or share administrative tasks in order to provide direct patient care or collectively manage crisis for a short period of time.
	Adjusting staff–patient ratios	Adapt and flex rules for nurse–patient ratios in intensive care. For example, senior staff may sometimes care for two level 3 patients. Use of students and healthcare assistants (supervised by a more senior staff member) to make sure all patients have a dedicated member of staff.
	‘Cohorting’ patients in the unit by acuity	Patients are moved around the unit so that the most critically ill patients are concentrated in an area with the most experienced staff, making care for patients more efficient and manageable for the whole team.
**Prioritise demand**		
	Prioritising patients within the unit by urgency and by who would benefit most	Prioritise patients and tasks by urgency. For example, physiotherapists had their own loosely held criteria of who to prioritise based on body area (eg, chest patients) and procedure (eg, tracheostomy).
	Transferring or relocating patients with less need	Discharge patients early to the ward when ordinarily they would be kept an extra day for reassurance. Outreach teams asked to follow-up on the ward.
	Temporarily stopping or delaying some activities/types of care	Delay or suspend some tasks and activities such as tasks relating to patient comfort such as washing and activities such as staff education and training. Tasks that were occasionally delayed temporarily included ward rounds, taking patients outside or talking to families.
**Adapt ways of working**		
Adapt communication	Increasing communication with other teams to coordinate faster admissions and discharges	Increase communication with outreach, operations and duty manager teams at times of pressure, to coordinate patient admissions and discharges with multiple areas of the hospital, including surgery, the emergency department and the wards.
	Use of instant communication to troubleshoot problems	Regular use of WhatsApp, emergency Microsoft Teams meetings and other means of instant communication to request urgent support or updates.
	Boards for monitoring and communication	An interface to monitor beds and demand during COVID-19, which was carried forward into routine practice to identify units under pressure for beds.
Adapt leadership	Greater presence ‘on the shop floor’	Be more visible, which often involved patrolling the unit to monitor, check-in and support where needed. Doctors use mobile computers on wheels to maximise efficiency of administrative work on the ward and to be immediately available in the ICU to support nurses.
	Using networks to advise and help	Knowing who to call in a crisis, with individuals relying extensively on personal contacts across the hospital.
	Providing additional support to staff and opportunistic teaching	Ensure staff take breaks, eat and monitor fatigue and well-being (eg, hot debriefs, welfare checks). Create learning opportunities where possible on the day, such as impromptu bedside teaching.
	Stepping back to have better awareness of the situation	Step away and find a quiet undisturbed place for a moment to gain a better overview of the situation and understand how best to prioritise and delegate tasks.
	Adjusting and communicating goals for the system	Call for a brief huddle to clarify aims or refer to contingency plans, including reallocation of roles, when an uncommon event occurs (eg, paediatric admission).
Adapt teamwork	Increasing collaboration and asking for help	Junior members of the team encouraged to ask for help and support, for example, transfer task to senior staff or delegate to healthcare assistants as appropriate.
	Task shifting between professions	Doctors assist with turning patients or cover breaks and nurses are asked to be involved in ward rounds. Physiotherapists monitor patients when nurses are unavailable or during emergencies (eg, cardiac arrest).
	Adjusting or making clear allocation of roles	Clear allocation of tasks, emphasising their priority and providing a timeframe when pressures are high.
	More use of confirmation and closed-loop communication	Increased use of closed-loop communication as a checking mechanism (read-back) to confirm instructions have been listened to and understood.

ICUsintensive care units

Box 2 **On-the-day adaptations in intensive care**

**Flexing resources to provide safest care within the available resources**

“We have a cardiac ICU, so at weekends they’re more helpful than in the week because in the week, if we’re transferring to cardiac ICU, it means cancelling a patient the next day. Whereas at weekend they’re not operating, we can utilise their beds occasionally, but we need to try and get them out by Monday so that they can carry on operating.” [Consultant]

**Taking a step back to prioritise and reprioritise**

“I guess taking five minutes to just reprioritise. Especially as a coordinator, you can get a bit swamped, but even at the bedside, just taking two minutes away is something I was taught when I started coordinating. Sometimes just go for a wee, regardless of whether you need to wee. Just go and lock yourself in a room, just shut the door, have a look at your handover sheet, what tasks still need doing, what haven’t I done, what haven’t I thought of? What still needs doing within the shift and how am I going to reprioritise that?” [Senior Nurse]

**Regular contact with operations teams to work with the wider system**

“We utilise the duty manager team, the operational team, who, if we are starting to feel pressure and we have patients who could be elsewhere, then when our pressures change, we’ll contact them again. And it may be that our risk has been increased because they’re looking at the hospital as a whole. We’ve admitted two patients in the last hour, which means we have no emergency space, whereas the surgical ward was full but actually now our risk is greater. So, they’ll take a patient back to surgery, which will reduce our risk down. So, we regularly contact. Throughout the day, we’re in contact with them as well to see whether they can create some flow back into the hospital again.” [Deputy Matron]

**Providing additional support to staff and consolidating learning**

“It’s about making sure that staff understand that I don’t like to put their head in the lion’s mouth, unless they’ve got a big helmet on. Have you got what you need? And some of it is emotional, but some of it is about making sure that they’re equipped adequately… It’s also celebrating when things are done really well. So if we come away going, that really went well, it’s saying to the team, why do you think that particular arrest went really well, and the other ones didn’t? So that we can also do that positive reporting.” [Outreach]

#### Flex resources

Decisions were often made on the day on how best to allocate the resources available to relieve pressures and maintain safety. During day shifts, last minute shortages might sometimes be remedied by borrowing staff from other areas of the hospital (eg, surgery or education teams). During night shifts, when the workforce is significantly reduced, staff relied more on task-switching strategies (eg, doctors helping nurses to turn patients). Adaptive reallocation of staff and adjustments to nurse–patient ratios were also deployed throughout the day according to staff experience and patient acuity. Participants spoke about continually assessing the safest place for patients at any given time, looking at the hospital as a whole and holding, transferring or relocating patients if pressures were high in the unit. There was a close collaboration within one group of hospitals who constantly monitored acuity and available resources across the network. However, it was understood that all possible avenues within a hospital would be exhausted before transferring a patient elsewhere.

#### Prioritise demand (patients and tasks)

Patient care was prioritised by clinical urgency based on clinical judgement and experience. This was especially difficult for more junior members of the team who were less able to judge which tasks were essential and which could potentially be delayed or missed. On busy days, some activities were deprioritised. For example, washing patients or education/training for staff would be postponed or cancelled so that necessary clinical care could be given.

#### Adapt ways of working

Clinical leadership and effective communication and teamwork are vital in intensive care at any time, but particularly important when care has to be constantly adapted to meet demand and manage risks. At times of high pressure, leaders spent more time on the unit to provide support at the bedside, identify emerging problems or tasks being missed. Leaders also ensured that staff took breaks and had food. Doctors who were new to the senior role on the unit were learning who in the hospital to call for support in a crisis.

Leaders of teams emphasised that careful listening and absolute clarity of communication was vital when under pressure—verbalise your own understanding of the patient and listen carefully to what others are saying. A senior leader explained that they would deliberately speak in a way that conveyed a sense of calm, speaking more slowly to reassure others that everything was under control. Methods of instant communication (eg, WhatsApp) were heavily relied on in these circumstances for quick updates or request urgent support.

### Deploying a portfolio of strategies

We have described and classified a considerable number of strategies, which might imply that clinical teams select particular strategies to employ at any one time. However, clinical leaders most often employ a variety of strategies in combination to dynamically manage pressure (box 3), though the approach varies considerably between individuals, teams and between hospitals. Furthermore, leaders are also having to decide which rules can be broken or temporarily suspended (eg, nurse–patient ratios) and which aspects of care can be sacrificed (eg, patient comfort) in order to manage risks and provide the best care possible within the available resources.

Box 3 **Case study. Dynamic use of strategies when demand exceeds resources (the sliding tile puzzle)**

“You’ll get requests for admission, should we bring this patient to the ICU, and then you’ve got to talk to the nurse and see if you’ve got a physical bed available and, if there is, is that staffed? If it’s not staffed, can we rearrange the patients on the ICU to optimise the nurse utilisation?

And sometimes that might involve doing things like saying, look, can you phone infection control and see if any of these patients can be de-isolated, or maybe what we’ll do is we don’t really believe this isolation is terribly important, so even though it’s strictly by the rules to keep them, we’re just going to break that because that’s not important.

You may have people who you may discharge slightly earlier than you would otherwise. Some people, you say, well, maybe I’ll keep them an extra 12 hours just to be safe, and you’ll say, look, I’m going to forego that extra bit and I’ll just use the outreach team to follow them up on the ward and just deal with it that way.

Or, for instance, if it’s an admission from another hospital, of course you can just say, no, we can’t, there’s no room at the inn, try someone else. Or can we delay the admission? If it’s a ward patient, maybe we can keep them on the ward and use outreach to follow them up. It kind of depends case by case on how badly does, if you like, the incoming patient need the ICU compared to the patients already on the ICU, compared to can we stretch the nursing ratios? That’s the other thing.

Are there a couple of patients where, actually, strictly speaking, you need one to one, but actually they’re pretty well, and why don’t we do them one to two? And they are very specific to the patients, I would say. I guess, I haven’t really thought about it, there must be some sort of broadly, loosely held kind of idea of the order in which the rules might be broken.

For instance, if we really overflow, then of course you can say, well, we’ll keep them in the theatre recovery, for instance, and nurse them there, if physical beds were a problem.” [Consultant]

## Discussion

The principal source of pressure identified in ICUs is a shortage of staff with the necessary skills and experience to be able to cope with the increased numbers and complexity of patients they were receiving, which had a knock-on effect on numbers of open beds available, staff workload and patient flow. Staff deploy a portfolio of strategies to respond to these pressures, with some deployed in advance of anticipated pressures and some on the day to manage immediate pressures. Clinical staff described a wide variety of anticipatory strategies to increase resources, control demand and adapt the way in which care was delivered. Flexing resources on the day was widely used, with many examples of task shifting between different job roles and levels of seniority. Coordinating with other areas of the hospital and use of outreach teams were vital ways in which ICUs could discharge early or delay admissions to manage demand.

A particular challenge for clinical leaders was to minimise the burden on the reduced senior staff available to supervise an inexperienced junior workforce. Adapting to a less experienced workforce required more senior presence, increased directness and clarity of communication and the development of simpler standardised protocols to cover the basics of care. Clinical leaders needed to provide much more guidance on prioritisation of patients and be explicit about which tasks were safety critical and which could be delayed or missed. However, patients were nevertheless sometimes being cared for by nurses without the expected levels of skills and experience. Effective supervision in a variety of contexts has been shown to enhance safety when patients are cared for by less experienced staff.^18 19^

Adaptive strategies almost always come at a cost, and some strategies cannot be used indefinitely without damaging the service in the longer-term.^7^ There is a risk that temporary adjustments become long-term normalised deviations from best practice,^20^ leading to a gradual but cumulative degradation of the system. For example, some ICUs deploy clinical skills practitioners, whose primary role is education and training, as a buffer when staff shortages occur. Clinical skills practitioners are experienced nurses who are highly capable at providing direct patient care, but the longer-term impact of this reallocation is a reduction in clinical skills training and a reduction in the overall skill level.^15 21^ This highlights the need to clearly identify adaptations and set limits on the time that they can be employed.^5 14 20^

### Taking what works: evolving through adaptive responses to pressure

Although not the focus of the study, the timing of the interviews meant that many interviewees spoke about adaptive strategies deployed during the COVID-19 pandemic, which had been adopted into everyday practice.^7^ Experience during the pandemic had highlighted the value of safety huddles and daily sitreps at times of high pressure. The pandemic had also led to new ways of monitoring patient status and staffing pressures, which enabled senior staff to control demand and flex resources more efficiently.

Considerable efforts had been made to simplify and standardise clinical protocols to make them more accessible to the junior staff with increased responsibility for very sick patients. The precise formulation of rules and standard operating procedures has been a longstanding concern in other high-risk industries.^22^ Standardisation in the care of complex patients must be approached cautiously and an insensitive demand to follow guidelines in all circumstances can indeed be detrimental to patient safety.^23^ However, articulating and explaining the routines used by expert clinicians is a powerful means of supporting less experienced staff and enhancing the safety of care.^24^

Increased pressures in this environment has fostered a greater understanding of the need for support for intensive care staff due both to the inherently stressful nature of the work and to the increasingly frequent but inevitable departure from basic professional standards.^3 25^ Some departures from standards are often accepted as necessary in the short-term, but in the longer-term, staff may experience distress and moral injury from not being able to meet personal and professional standards.^26^ Psychologist and well-being teams have grown considerably in recent years with dedicated support for intensive care staff in all four hospitals.^26 27^ There is a critical role for leaders, both executive and clinical, in discussing such compromises openly and supporting teams faced with unenviable decisions. The risk of moral injury will be less if such decisions are seen as a necessary collective decision rather than an individual personal failing.^26^

### Strengths and limitations

We purposely sampled four hospitals that were diverse in size and location, and across different professions, to provide a breadth of perspectives regarding the nature of pressures experienced and strategies used. Most participants were relatively senior, in that they were able to initiate system-level adaptations,^28^ although of course more junior staff are adapting every day as well. Our choice of interviews for data collection meant that participants could discuss and reflect on adaptive strategies, which methodologies such as ethnography used in other similar studies are less suited to; conversely there may be some strategies that could be observed through ethnographic methods but are not easily describable by participants in interviews which we may have missed. Conducting the study relatively soon after the COVID-19 pandemic, which disproportionately affected intensive care staff more than other hospital settings, might have had some bearing on the types of pressures and strategies described. We were also not able to evaluate the effectiveness or impact and unintended consequences of the various strategies, which needs to be a priority for future research.

### Future research on adaptive strategies

The taxonomies of pressures and strategies previously developed by the research team^8^ provided an effective framework to categorise the results of this research: the framework was able to capture the key strategies and pressures described by participants. Future research is needed to test whether the framework works well in other clinical contexts such as primary care or mental health settings and also in other countries where healthcare team cultures and ways of working can be very different.^8 29^ Research is also needed to understand how certain strategies can be linked with certain pressures, building on recent work by Sanford *et al*.^6^

Within intensive care specifically, further research is needed to explore the effectiveness of different combinations of strategies. There may be certain strategies or combinations of strategies that are better than others or have differential trade-offs and impact on safety, staff well-being, patient flow and patient experience.^2 5 6 9 30^ A strategy may for instance reduce risk for patients but increase burden on staff: this could be explored further through vignette-based studies, as has been explored by others.^2^ Programmes could be established to test and evaluate different combinations of strategies in response to familiar pressures experienced in intensive care.^5^

There is also a need to assess the benefits and risks of specific adaptive safety, such as discharging patients from ICU directly home.^31 32^ Outreach services and acute hospital at home teams were increasingly being used to provide follow-up support for patients at home. Patients were carefully selected based on the complexity of their condition and the support from both family and professionals at home. However, if this becomes a more widely used approach, there is a risk of over-burdening families and exporting risk out of hospitals and into the home.^33 34^

### Training in adaptive strategies and working under pressure

The use of adaptive strategies is often learnt experientially on the job, highly individualised and not explicitly taught.^1 14^ The strategies described here could help clinicians and managers respond to similar pressures, providing a portfolio of coordinated strategies that clinical teams could use or develop for their own contexts.

Many ICUs already have some form of simulation and skills training, which would naturally lend itself to training in team and system-level adaptations. For example, scenario-based teaching around managing competing demands could help to reduce stress and uphold safe practices when individuals have to make strategic decisions quickly in pressurised situations.^35 36^ Wider, more formal training programmes will require organisations, and indeed regulators, to explicitly acknowledge the difficulties of maintaining standards of care when under pressure and see such training as a necessary form of proactive risk management. Future work will explore what this type of training might look like and how it could be organised, with attention given to efficacy, trade-offs and implications of a menu of strategies.

## Conclusion

Intensive care is a highly pressurised environment which requires frequent adaptation to maintain patient safety. While these adaptive strategies are necessary and aimed at providing better care in the short-term, they also involve substantial risks to patients and potential longer-term degradation of standard of care. Our aim in this study however is not simply to point to the adaptations but to pave the way for a more open, transparent, coordinated and time-limited approach to adaptations. The strategies currently employed are highly variable, often improvised and often not shared within the clinical team. We believe that patients will be safer if we develop prepared and coordinated strategies, where the team has agreed an approach for managing risk under pressure.^5^

## supplementary material

10.1136/bmjqs-2024-017385 online supplemental file 1

10.1136/bmjqs-2024-017385 online supplemental file 2

## Data Availability

No data are available.
