# Supplementary material for: Strategies for adapting under pressure: an interview study in intensive care units
Source: BMJ Qual Saf. 2024 Aug 23;34(2):e017385. doi: 10.1136/bmjqs-2024-017385 (PMC11874268; doi:10.1136/bmjqs-2024-017385)
Supplement: online supplemental file 2 [file bmjqs-34-2-s002.pdf]

Supplementary File 2: Adaptive Strategies in ICU - NVivo Codebook

This file shows the code book used for analysing the interviews. The code book is based on the taxonomy of pressures and strategies previously published in *Page B, Irving D, Amalberti R, et al. Health services under pressure: a scoping review and development of a taxonomy of adaptive strategies. BMJ Quality & Safety Published Online First: 29 November 2023.*

Name	Description	Files	References
**Context**		1	1
About the interviewee		20	65
Descriptions of the clinical setting		17	46
**Pressures**		4	5
Demand exceeding capacity		7	8
Patient demand		1	1
High patient acuity		16	40
High volume of patients		10	15

Name	Description	Files	References
Patients' and families' concerns & expectations		5	11
Resource pressures		1	1
Lack of space or space not fit for purpose		12	24
Shortage of bed availability		11	17
Shortage of medications, supplies, equipment		4	8
Shortage of staff numbers eg. vacancies, sickness, strikes		19	103
Shortage of staff with the necessary skills		15	68
Staff not pulling their weight		1	3
Wider contextual pressures		0	0
Organisational pressures	Funding, pressures in other parts of the system	10	29

Name	Description	Files	References
Strikes		2	3
Socio-Economic pressures	e.g. Covid pandemic, cost of living crisis	11	19
Problems with system performance		0	0
Patients		1	1
Delays in patients accessing or receiving care		3	5
Missed care or errors		13	26
Overcrowding		1	1
Problems with patient flow		8	18
System and staff		2	3
Difficulties monitoring the situation		4	7
Lack of buffer for ad-hoc requests or unplanned work		1	1

Name	Description	Files	References
Problems with cooperation or communication		10	16
<u>Difficult working conditions</u>		0	0
Staff-team dynamics		3	5
Interpersonal difficulties		8	19
Lack of support for staff		8	29
Staff stress or poor wellbeing		16	58
Team changes		10	25
Workload pressures		0	0
Difficulties prioritising workload		10	15
High or increased interruptions		6	6
High workload, or increased complexity of workload		16	63

Name	Description	Files	References
Workload to train and support new nurses		3	4
Time pressures		7	11
**Strategies**		0	0
Anticipatory strategies		0	0
Plans for managing workload		0	0
Efficiency strategies		5	10
Doing tasks ahead of busy time		1	1
Scheduling to maximise use of limited resource		1	1
Use of technology and automated systems		7	14
Forward planning		5	8
Adapting protocols		6	15

Name	Description	Files	References
Anticipatory prioritisation		2	2
Contingency planning		10	28
Monitoring or co-ordination strategies		2	2
Centralised structures for co-ordination		3	4
Having an up-to-date knowledge of resources and demand		6	14
Planned meetings for monitoring the situation and communicating plans		16	43
Getting the nurses to join the doctors on the ward round- query teamwork		1	1

Name	Description	Files	References
Staff support initiatives		13	30
Support and education for overseas nurses		4	7
Resources vs demand		1	1
Control demand		0	0
Discharging or transferring patients		7	21
Suspending or restricting services or procedures		7	9
Increase resources available		1	1
Create more space or repurpose space		5	6
Improve skillmix		14	34
Support and education for overseas nurses		5	8

Name	Description	Files	References
Increase staff		13	29
New job role or new team		2	2
Increase supplies		2	3
Making managers or politicians aware of pressures		6	11
Open more beds or services		3	5
On-the-day adaptions		7	9
Adapting ways of working		1	1
Communication		13	34
Boards for monitoring and communicating (e.g. patient status)		3	3

Name	Description	Files	References
Encourage team to document things		1	1
Increased communication (e.g. additional ad hoc meetings to monitor the situation)		7	10
More reliance on face-to-face communication and handwritten notes (less reliance on electronic communication)		2	2
Other means of quick communication, e.g. WhatsApp groups		5	9
Simplifying information		1	2
Communication needs to be clear		6	9
Leadership		12	46

Name	Description	Files	References
Adjusting and communicating the goals for the system		7	11
Making decisions based on current information		2	5
Explaining why something needs doing to staff and pre-empting the next steps		3	6
Knowing your team		12	27
Making sure you are aware of your team's skills		5	5
Leaders spend more time on the 'shop floor'		8	17
Learning for next time		1	2
Providing support to staff		19	72

Name	Description	Files	References
Ensuring people have their breaks and don't feel guilty		7	9
Helping people feel comfortable about asking for help		6	13
Remaining calm under pressure		7	12
Stop operations and regain awareness of situation		1	1
Use of networks		7	7
Teamwork		1	1
Checking mechanisms		4	4
Checking work has been done and not just assuming		2	3

Name	Description	Files	References
Closed loop communication		2	3
Clear allocation or adjustments to the allocation of roles		9	14
Collaboration strategies and asking for help from others		5	6
Ensuring staff don't take too long breaks when busy		1	1
Getting the nurses to join the doctors on the ward round- query teamwork		1	1
Interdisciplinary working		3	4
Linking with other units		3	6

Name	Description	Files	References
Making sure you are aware of your team's skills		0	0
Not having a break		2	2
Pre-empting things		1	1
Sending outreach to see a patient in A&E to assess whether suitable for ICU		1	1
Silo working		1	1
Speaking up when you don't know		1	1
Support for less experienced staff		2	2
Teamwork	Strategies relating to team work	13	41
Use of protocols and guidance		8	16

Name	Description	Files	References
Reflecting at the end of the busy day		3	5
Flexing the use of resources		3	4
Use of existing drugs and equipment		0	0
Borrowing resources from other units		0	0
Use of similar drugs or equipment		1	1
Use of existing space and beds		4	7
Creating temporary holding spaces for patients		2	2
Placing patients or providing care in non-standard areas		0	0

Name	Description	Files	References
Transferring or relocating patients based on need		6	15
Change in the use of critical care beds based on patient demand across hospital		2	2
Placing patient in another related unit		1	1
Use of existing staff		3	8
Adjustments to staff-patient ratios		6	7
Flexing staff to address numbers or skill-mix (e.g. reallocating staff between units)		12	38
Managerial staff take on clinical roles		6	15

Name	Description	Files	References
Education team help on busy days		3	4
Staff work late		1	1
Staff staying late		5	9
Task-shifting or extension of responsibilities		5	9
Allocation of staff and task-shifting	Strategies relating to how staff are allocated and job roles	9	26
Nurse asking doctor to watch the patient for a few minutes		1	1
Prioritising demand		0	0
Prioritisation of workload		3	4
Prioritisation and reprioritising workload		9	49

Name	Description	Files	References
Prioritisation of workload or tasks		7	22
Prioritising and reprioritising patients		13	33
Temporarily stopping some activities or types of care, or delaying till later		8	15
**Education and learning**		1	1
Education and learning for others	Top tips for others to ensure pressures don't impact on safety and advice for others	14	40
How these strategies are developed	Descriptions of how interviewee learnt or developed the strategies they use	0	0
Formal teaching		13	26
Informal teaching		2	2
Learning from experience		16	40
Mentoring or coaching		6	11

Name	Description	Files	References
Other		5	12
Seeing what others are doing		10	12
Simulation		3	5
How to teach these strategies	Thoughts and advice on how to teach these strategies	4	6
Bed-side teaching		4	8
Formal teaching		4	8
Mentoring or coaching		4	8
Other		5	8
Protocols		3	5
Simulation and scenario-based		5	19
**Impact**		0	0
Impacts of pressures on patients and families		15	45
Impacts of pressures on staff		15	42

Name	Description	Files	References
Impacts of pressures on the wider system	This includes impacts on safety, impacts on other parts of the health system such as A&E, surgery or discharges	10	18
**Main objective**		17	28
**Sage advice**		12	23
Leave personal issues at home		1	1
Useful vignettes on pressures		7	12
